# Fruit Battery Method for Oil Palm Fruit Ripeness Sensor and Comparison with Computer Vision Method

**DOI:** 10.3390/s20030637

**Published:** 2020-01-23

**Authors:** Nor Aziana Aliteh, Kaiko Minakata, Kunihisa Tashiro, Hiroyuki Wakiwaka, Kazuki Kobayashi, Hirokazu Nagata, Norhisam Misron

**Affiliations:** 1 Faculty of Engineering, Shinshu University, 4-17-1 Wakasato, Nagano 380-8553, Japan; aziana.teh@gmail.com (N.A.A.); mnktsilkukoika@gmail.com (K.M.); wakiwak@shinshu-u.ac.jp (H.W.); kby@shinshu-u.ac.jp (K.K.); 2 Global Education Center, Shinshu University, 3-1-1 Asahi, Matsumoto 390-8621, Japan; hnagata@shinshu-u.ac.jp; 3 Faculty of Engineering, Universiti Putra Malaysia, Serdang, Selangor 43400, Malaysia; norhisam@upm.edu.my

**Keywords:** oil palm, moisture content, fruit battery method, load resistance voltage, color feature, SVM

## Abstract

Oil palm ripeness’ main evaluation procedure is traditionally accomplished by human vision. However, the dependency on human evaluators to grade the ripeness of oil palm fresh fruit bunches (FFBs) by traditional means could lead to inaccuracy that can cause a reduction in oil palm fruit oil extraction rate (OER). This paper emphasizes the fruit battery method to distinguish oil palm fruit FFB ripeness stages by determining the value of load resistance voltage and its moisture content resolution. In addition, computer vision using a color feature is tested on the same samples to compare the accuracy score using support vector machine (SVM). The accuracy score results of the fruit battery, computer vision, and a combination of both methods’ accuracy scores are evaluated and compared. When the ripe and unripe samples were tested for load resistance voltage ranging from 10 Ω to 10 kΩ, three resistance values were shortlisted and tested for moisture content resolution evaluation. A 1 kΩ load resistance showed the best moisture content resolution, and the results were used for accuracy score evaluation comparison with computer vision. From the results obtained, the accuracy scores for the combination method are the highest, followed by the fruit battery and computer vision methods.

## 1. Introduction

Palm oil is the most productive vegetable oil in the world. It accounts for about 30% of the total production of vegetable oils and fats in the world. Palm oil has proven to be useful and is used in various products such as soap, margarine, cosmetics, and surfactants [1].

Currently, traditional human graders play an important role in distinguishing oil palm ripeness in plantations. During the pre-harvesting stage, human graders evaluate the oil palm fresh fruit bunches (FFBs) based on the number of detached fruits that fell to the ground and the surface color of oil palm FFBs to determine their ripeness stage [2]. For the post-harvesting stage, a human expert inspects the color of the oil palm FFBs’ surface to reject unripe and over-ripe FFBs so that they do not proceed to the oil extraction process [3]. According to Siregar [4], oil extraction rate (OER) decreases by 0.13% if unripe fruits are present, up to 1% during oil extraction. Furthermore, the National Economic Advisory Council (NEAC) in Malaysia reported that if OER rises by 3%, it brings economic profit of RM 8 billion [5]. In other words, if there is a 0.13% drop in OER due to misjudgment, it will cause the equivalent of RM 340 million in economic loss. Therefore, it is essential to use an automated detection classification method to prevent losses and increase productivity.

Several detection methods have been introduced, and computer vision is one of the main methods that are widely used for this application. Other than red-green-blue (RGB), color evaluation methods are also used such as hue, saturation and intensity (HSI) [6]. Research conducted by Cherie et al. [7] under three spectrum regions tested ultraviolet, visible, and near infrared to evaluate the oil palm FFB harvesting decision. Besides that, Hazir et al. [8] introduced a multi-parameter fluorescence sensor to investigate the potential of flavonoids and anthocyanins parameters as a predictor to classify the degree of oil palm FFB ripeness.

Conventionally, the computer vision evaluation is paired with the automated artificial intelligence system by computer to accurately grade the oil palm fruit ripeness stages. The methods mentioned are based on image processing, which requires complicated procedures and strictly controlled conditions for measurement, such as no environmental light and constant settings of measurement devices. Therefore, they require image color correction calibration, which uses an image processing software and manually picks up pixel values on a color chart that is placed next to a target object in an image.

There are also various microwave sensors that have been tested for the same purpose, as Ahmad et al. [9] suggested, such as coplanar and microstrip sensors. The measurement system was tested with a microstrip ring resonator that operated between 2.2 and 3 GHz on oil palm FFB seeds and fruits with various maturity stages. However, the fruit sample preparation is very time-consuming, since the oil palm FFB mesocarp need to be separated from its seeds, and the mesocarp is mushed into semi-solid sample.

Furthermore, the new inductive method was also proposed with a different structure: circular, single, dual, and triple coil structure [10]. This sensor was developed based on the fact that the resonant frequency of the sensor increases as the fruit ripens and inversely, the capacitance value decreases as the fruit ripens [11]. However, these inductive methods are time-consuming and require specific equipment for measurement and analysis.

For this study, a fruit battery is proposed as new detection method together with computer vision. The fruit battery method measures the load resistance voltage when copper and zinc electrodes are embedded in the oil palm fruit. The value of load resistance voltage is different between unripe and ripe fruit principally due to the presence of ions in the form of fruit moisture content, which serves as the electrolyte. The advantage of the fruit battery method is that it is possible to construct a simple and inexpensive detection device, since ripeness can be determined by simply measuring the load resistance voltage. In a previous study, suitable electrode conditions for the fruit battery method for depth and distance were discovered. It was concluded that 3 mm depth and 2 mm distance between electrodes are the best parameters when evaluating load resistance voltage results and shape of oil palm fruit [12].

The objective of this study was to determine the best load resistance value that produces the highest sensitivity for the fruit battery method when tested with fruit sample at different maturity stages. Besides that, this study also aimed to evaluate the accuracy score for fruit battery and compare it with the computer vision method’s accuracy score applied to the same fruit sample.

## 2. Materials

### 2.1. Sample Preparation

**Figure 1:**
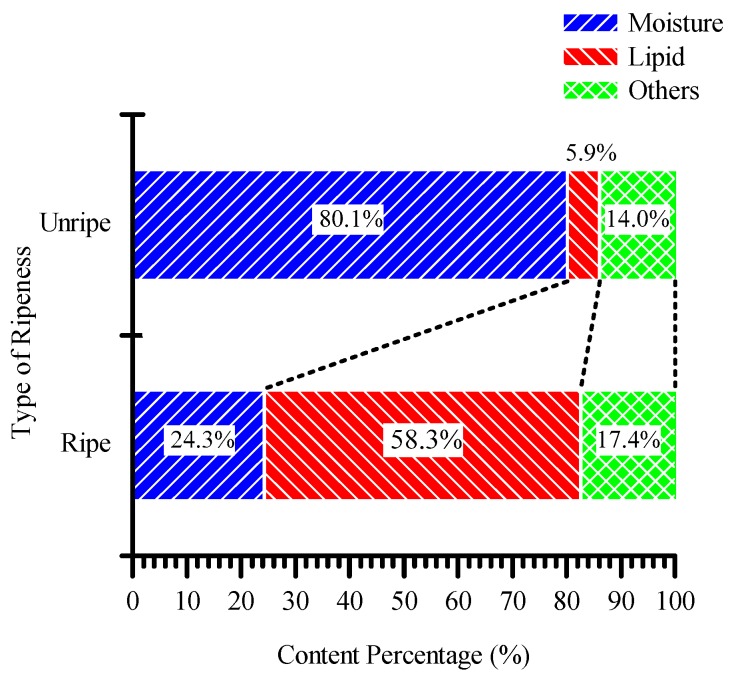
The composition of unripe and ripe fruit [11].

The sample collection and experiment were conducted for three months from 15 September 2017 to 15 December 2017. The location where samples were obtained is at the Universiti Putra Malaysia oil palm plantation. Oil palm ripeness is determined by moisture contents, as shown in Table 1. From Table 1, when moisture content is 30% or less, it is labeled as “Ripe”. Meanwhile, when moisture content is between 30% and 53%, it is labeled as “Under-ripe”, and moisture content of more than 53% is labeled as “Unripe”. Table 1 is derived from the fact that FFBs that have 30% moisture content have the maximum oil content [4], and FFBs start to mature when moisture content is estimated at 53%, as shown in Figure 1 [11]. From Figure 1, oil palm fruit mainly consists of moisture and lipids. The percentages of moisture and lipids in unripe fruit are 80.1% and 5.9%, respectively, while in ripe fruit they are 24.3% and 58.3%, respectively. It can be seen that the lipid content increases, and moisture content decreases, as the fruit matures.

### 2.2. Moisture Content Determination

The oil palm fruit sample tested underwent moisture content determination measurement. The determination evaluation was performed on the day of sample collection after measurements were taken. An infrared moisture meter FD-610, Kett (Kett Electric Laboratory, Tokyo, Japan) was used to measure moisture content. The measurement heating temperature condition was 105 °C with 60 min drying time.

## 3. Methodology

A total of 52 fruit samples were collected and used for this experiment. Among 52 fruit samples collected, 21 ripe, 15 under ripe, and 16 unripe fruits were identified according to their moisture content from Table 1. Photographs were taken of all samples using an augmented reality (AR) marker, and they were then tested with the fruit battery method. Lastly, moisture content was determined using the infrared moisture analyzer.

Firstly, load resistance determination was tested using only ripe and unripe fruits. Twenty-one ripe and 16 unripe fruits were tested with load resistance ranging from 10 Ω to 1 MΩ to obtain the differences between ripe and unripe samples and observe the best load resistance to be chosen for moisture content resolution analysis. Moisture content resolution analysis used all 52 fruits collected to identify the best load resistance that produced the highest sensitivity. The best load resistance results were used for accuracy score evaluation together with the computer vision.

For the computer vision method used in this study, we used an automatic and low-cost color correction method using augmented reality (AR) technology [13] and classified samples with the machine learning algorithm. The proposed method requires no strict calibration and adjustment at the measurement stage and automatically picks up pixel values of a color chart with an AR marker based on the relative position from the marker. For the present study, the accuracy scores obtained were saturated between 80% and 90%. This is due to the FFB surface color that exhibits similar color distinctiveness even though their ripeness stages are not distinct. Therefore, in order to improve its accuracy, an add-on feature of color feature identification was used simultaneously to increase the accuracy of classifying oil palm ripeness stages.

The following subsections explain further the computer vision and fruit battery methods applied in this study.

### 3.1. Basic Concept of Fruit Battery Method

**Figure 2:**
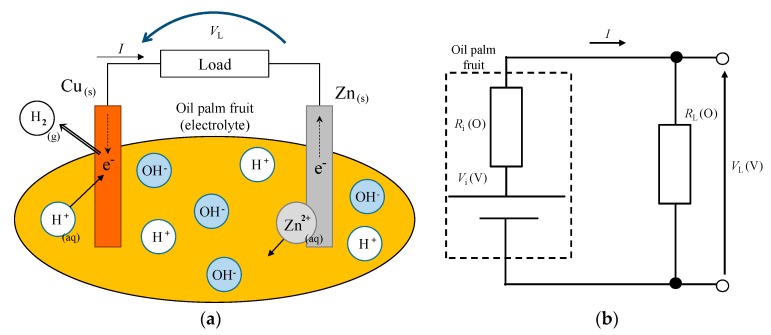
(**a**) Schematic diagram of the fruit battery and (**b**) simple equivalent circuit of the fruit battery.

This paper proposes the fruit battery method to distinguish oil palm fruit ripeness by utilizing the principle of the fruit battery. Figure 2a shows a schematic diagram illustrating the principle of a fruit battery using an oil palm fruit. Equations (1) and (2) express the chemical equation of the fruit battery. A fruit battery basically generates electromotive force when two electrodes with different standard electrode potential are pierced through the fruit surface. Figure 2a shows that when a copper and a zinc electrode pierce through the fruit, the zinc atom undergoes oxidation reaction where it loses an electron, since zinc is located higher in electrochemical series than copper. The copper electrode accepts the electron from the zinc electrode, and the electron is combined with a positive hydrogen ion from the fruit, producing a hydrogen molecule as shown in Equations (1) and (2). The movement of an electron generates current flow, thus producing electricity and behaving like a battery, hence the name fruit battery.

Zn (s) → Zn^2+^ (aq) + 2e^−^ (1)

H^+^ (aq) + 2e^−^ → H_2_ (g) (2)

Figure 2b shows an equivalent circuit of a fruit battery. The equivalent circuit of the fruit battery can be expressed by an electromotive force *V*_i_ (V) and an internal resistance *R*_i_ (Ω). Thus, the ripe and unripe oil palm fruit internal resistance is high or low depending on the fruit’s moisture content. The differences in internal resistance causes the load resistance voltage *V*_L_ (V) differences, as shown in Equation (3). The fruit battery method aims to detect the difference in electrolyte load resistance voltage between unripe and ripe fruit.

(3) $$V_{L}=\frac{R_{L}}{R_{i}+R_{L}}V_{i}$$

### 3.2. Selecting Load Resistance Value

To determine a suitable load resistance for distinguishing oil palm ripeness stages for the fruit battery method, two experiments were conducted. The first experiment aimed to calculate the differences of the load resistance voltage. The load resistance voltage differences show the behavior of load resistance with oil palm fruit from different maturity stages with different moisture content. The second experiment aimed to derive the resolution of the estimated moisture content for the field test. The resolution of (A/D) converter and the value of slope of regression formula between the load resistance voltage and moisture content were derived to determine the moisture content resolution.

### 3.3. The Changing Rate of the Load Resistance Voltage

**Figure 3:**
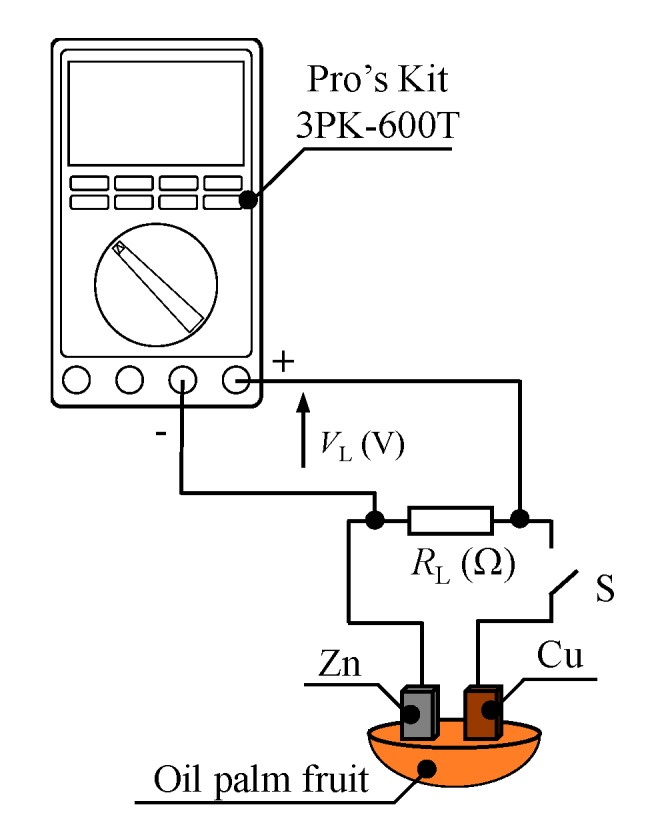
Fruit battery experimental setup.

Figure 3 and Table 2 show the experimental setup schematic and experimental condition, respectively. The load resistance voltage was measured using a digital multimeter Pro’s Kit, 3PK-600T (Prokit’s Industries, New Taipei City, Taiwan) when electrodes were embedded into oil palm fruit, as shown in Figure 3. Zinc and copper were used as the electrode, and the dimensions of the electrodes were 16 mm long, 6 mm wide, and 0.5 mm thick. As determined from a previous study, the best distance between the electrodes is 2 mm with 3 mm depth [12]. The total sample used was 37 fruits, where 21 and 16 fruits were identified as ripe and unripe fruit, respectively. In those conditions, the load resistance R_L_ (Ω) tested varied from 10 Ω to 1 MΩ.

In order to determine the value of load resistance that can effectively differentiate the value between unripe fruit and ripe fruit, the average load resistance voltage of both unripe and ripe fruit was calculated. The average load resistance voltage was calculated in order to determine the value of load resistance voltage that differentiates the value between unripe and ripe sample. Then, load resistance voltage differences percentage of the load resistance voltage between unripe and ripe fruit was calculated using Equation (4):

(4) $$|d_{VL}|=|\frac{V_{LRipe}-V_{LUnripe}}{V_{LUnripe}}|\times 100\%$$

where *d*_VL_ is the load resistance voltage difference between unripe and ripe, *V*_LRipe_ is the ripe fruit’s load resistance voltage, and *V*_LUnripe_ is the unripe fruit’s load resistance voltage. Large |*d*_VL_| means larger resolution, and this improves the load resistance sensitivity to distinguish the differences between ripe and unripe fruits.

### 3.4. Resolution of Estimated Moisture Content

To derive the resolution of the estimated moisture content, the slope value of regression formula was determined using least squares method. A total of 52 oil palm fruit samples were used in this experiment. The best value of load resistance that was determined from a previous experiment was selected for this experiment.

Then, a scatter plot between moisture content and the load resistance voltage was plotted, and the regression formula between them was derived. Next, the values obtained were compared to each other and evaluated. The load resistance voltage was measured three times and the results were averaged.

### 3.5. Computer Vision

**Figure 4:**
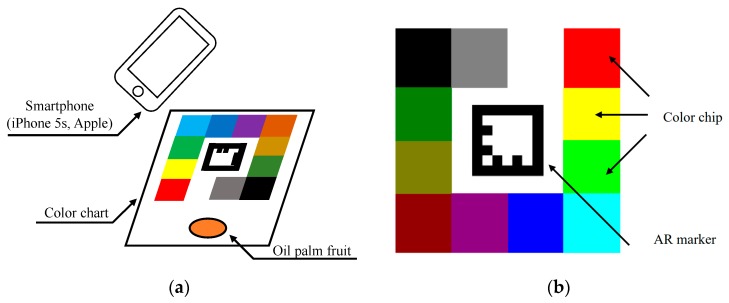
(**a**) Experimental setup of computer vision and (**b**) augmented reality (AR) marker-based color chart for automatic color correction.

For computer vision, a color chart and an oil palm fruit sample were taken together using a camera with a pixel resolution of 3264 × 2448 on a smartphone iPhone 5S (Apple Inc., Cupertino, CA, USA), as shown in Figure 4a. At this time, the picture was taken under a fluorescent light, and the color correction was conducted to decrease the influence of variation of photographing condition by using the color chart [14]. The color chart with AR marker was used to automatically correct the color condition of images. 

Figure 4b shows the AR marker based on color chart that has 16 color chips for color correction. The color chips are located around the marker, and each position of a color chip was automatically identified when the marker was detected. The proposed method requires a reference color chart image that is taken in the ideal lighting condition. Target images for color correction are images that include a target object, such as oil palm fruit and the AR color chart.

In the correction process, a transformation matrix (regression coefficient matrix) in Equation (5) was calculated by linear multiple regression analysis as the pixel values of the color chips were closest when using 16 RGB values from the color chips on the reference image and the target image. The color of the target image was changed by multiplying the transformation matrix a by the all pixel matrix C as shown in Equation (5). The advantages of the proposed color correction method are a reduction in the influence of the environmental lighting and a less complicated data acquisition procedure.

(5) $$\left[\begin{array}{c} C_{R}^{\prime }\\ C_{G}^{\prime }\\ C_{B}^{\prime } \end{array}\right]=\left[\begin{array}{cccc} a_{1} & a_{4} & a_{7} & a_{10}\\ a_{2} & a_{5} & a_{8} & a_{11}\\ a_{3} & a_{6} & a_{9} & a_{12} \end{array}\right]\left[\begin{array}{c} \begin{array}{c} C_{R}\\ C_{G}\\ C_{B} \end{array}\\ 1 \end{array}\right]$$

$C^{\prime }$: A corrected pixel value; C: An original pixel value; a: A transformation matrix.

**Figure 5:**
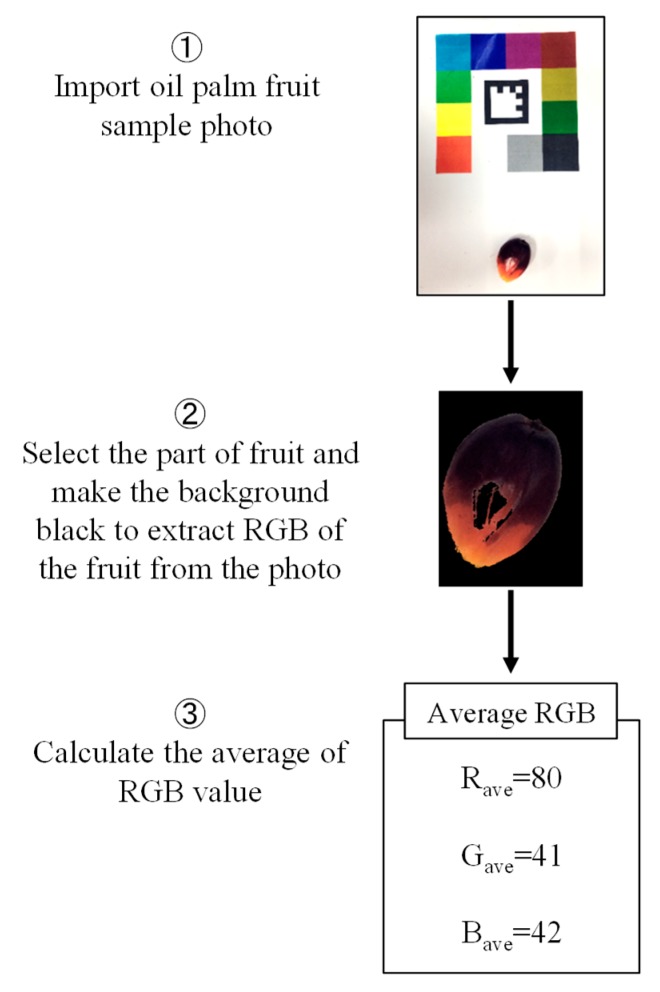
The flowchart of extracting color feature procedure.

After taking the picture, the average *RGB* value of oil palm fruit was extracted, as shown in Figure 5. To extract the average *RGB* value, the picture taken was imported, and the background was changed to black. In the background removal, a pixel value was set to 0 when the condition (*R*-value > 90, *G*-value > 90 and *B*-value > 90) is satisfied. Then, the average *RGB* value of the oil palm was calculated using Equation (6). The color feature R_ave_/G_ave_ extraction was done by using Numpy, a numerical calculation library in Python.

(6) $$\mathrm{Average}\;\mathrm{pixel}\;\mathrm{value}=\frac{\mathrm{Total}\;\mathrm{pixel}\;\mathrm{value}}{\mathrm{Total}\;\mathrm{number}\;\mathrm{of}\;\mathrm{pixel}}$$

We used the ratio R/G because we set the reference wavelength to increase the stability. In the field of remote sensing and plant physiology, the ratio of light intensity of wavelengths is often used to make vegetation indices such as normalized difference vegetation index (NDVI) [15] and green normalized difference vegetation index (GNDVI) [16].

### 3.6. Accuracy Scores

The accuracy score for three stages of ripeness was derived by support vector machine (SVM). Accuracy score was used as a metric to evaluate classification model in this study. Table 3 shows the experimental condition setup for SVM. From Table 3, the classification by SVM was performed with three features, that is: Color, R_ave_/G_ave_; fruit battery, *V*_L_; and combined computer vision with fruit battery method, *V*_L_-R_ave_/G_ave_.

The hyperparameters for the grid search are C = 1, 10, 100; Gamma = 1, 0.1, 0.01; Kernel = Linear, rbf; and the number of divisions of k-fold cross validation are 8. Hence, the combination produces 18 sets of accuracy scores where each feature is compared for the best score.

## 4. Results and Discussion

### 4.1. The Load Resistance Voltage Differences

**Figure 6:**
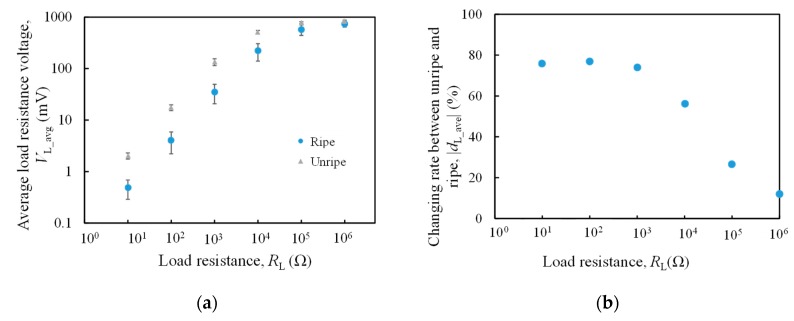
(**a**) The load resistance voltage of unripe and ripe as a function of load resistance and (**b**) the changing rate of load resistance voltage between unripe and ripe fruits.

Figure 6a shows the average value of the load resistance voltage when it changes. As shown in Figure 6a, in the range of 10 Ω to 10 kΩ, there is a tendency that the value of the unripe load resistance voltage is larger than ripe fruit. However, the difference between unripe and ripe does not occur at 100 kΩ and 1 MΩ. Figure 6a also shows the load resistance voltage differences between unripe and ripe fruit. 

From Figure 6b, 10 Ω is 76%, 100 Ω is 77%, 1 kΩ is 74%, and about 76% change of rate is obtained in the range of 10 Ω to 1 kΩ. For values larger than 1 kΩ, 10 kΩ is 57%, 100 kΩ is 27%, and 1 MΩ is 12%. When the load resistance increased, the change rate decreased. This is because the load resistance voltage is the ratio of load resistance and the internal resistance, as mentioned in Equation (4). The oil palm ripeness evaluation was performed by measuring the difference between internal resistance of unripe and ripe fruits. However, if the load resistance was too high compared to the internal resistance, the difference between unripe and ripe fruit’s internal resistance was too small to be detected.

From the results obtained, shown in Figure 6b, the changing rate of the load resistance voltage between unripe and ripe in the range of 10 Ω to 1 kΩ is the highest at about 76%, compared to the resistance values of 10 kΩ, 100 kΩ, and 1 MΩ tested. Hence, for the following section, the suitable load resistance was chosen from 10 Ω, 100 Ω, and 1 kΩ by calculating the resolution of estimated moisture content.

### 4.2. Resolution of Estimated Moisture Content

**Figure 7:**
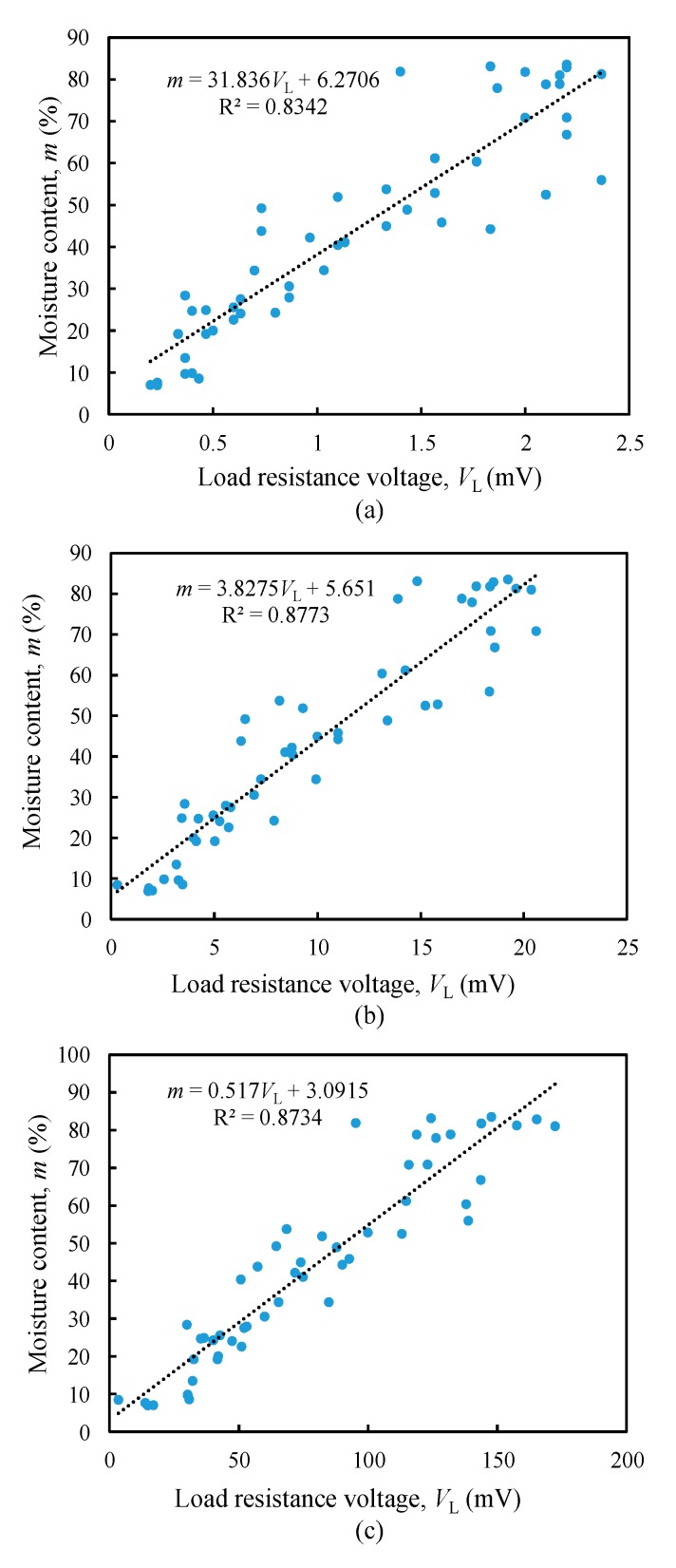
The prediction scatter plot between load resistance voltage and moisture content, (**a**) 10 Ω, (**b**) 100 Ω, and (**c**) 1 kΩ.

Based on results from Figure 6b, load resistance value from 10 Ω, 100 Ω, to 1 kΩ were tested, as shown in Figure 7. The moisture content varies directly with load resistance voltage. As the oil palm fruit ripens, the load resistance voltage decreases. Figure 7 shows the prediction scatter plot together with its regression equation for each resistance value tested.

Higher resolution means that the device is sensitive to detecting small change of the measurand in input. Thus, higher moisture content resolution with less than 1% can produce more accurate results with higher sensitivity. From Figure 7, it is observed that the 1 kΩ resistance gradient value is 0.517 %/mV, whereas 10 Ω and 100 Ω have resolution of moisture content exceeding 1% with 31.8%/mV and 3.83%/mV, respectively. Thus, 1 kΩ is the best load resistance value among them as it has the highest moisture content resolution.

### 4.3. Comparison Evaluation of Fruit Battery Method and Computer Vision

The results in the previous section emphasize fruit battery evaluation and moisture content determination and getting the best resolution out of the load resistance tested. The best load resistance of 1 kΩ was selected to be used for comparison and combining features with the computer vision. The fruit battery load resistance voltage was measured with the electrode distance and depth of 2 mm and 3 mm, respectively, with the value of load resistance of 1 kΩ. The average load resistance voltage of three measurements is used for data evaluation.

Table 4 shows the accuracy scores and standard deviation with its corresponding cost parameter, gamma, and kernel for the fruit battery method. The maximum accuracy scores are 0.9038 (90.4%). However, there are three results with 0.9038 accuracy score. The first result has cost parameter = 1, gamma = 1, standard deviation = 0.0712. The second results have the same standard deviation as the previously mentioned parameter, but with cost parameter = 10 and gamma = 0.1. The third has cost parameter = 100 and gamma = 1, but the standard deviation is 0.766. The best value among these three same accuracy scores is the standard deviation with the lowest value, since low standard deviation means that the data are spread out closer to the mean [17].

Table 5 shows the SVM analysis results for the computer vision method, where the highest accuracy score is 0.8654 (86.5%). This score has cost parameter = 1, gamma = 1, kernel = rbf with standard deviation = 0.0925.

Table 6 presents the results for the fruit battery and computer vision combination accuracy score. The maximum accuracy score obtained for the combination scores is 0.9423 (94.2%) with cost parameter = 10, gamma = 0.1 and standard deviation = 0.0804.

Table 7 shows the summary of maximum accuracy score and its standard deviation when each feature quantity is used: fruit battery, computer vision, and combination. The accuracy score is 90.4% for the fruit battery method *V*_L_, 86.5% for the color feature R_ave_/G_ave_, and 94.2% for the combined feature *V*_L_-R_ave_/G_ave_. The standard deviation is 0.0712 for load resistance voltage, 0.0925 for color features, and 0.0804 for combined features. The combination of the fruit battery method and computer vision method to classify the oil palm fruit ripeness stage results shows accuracy score improvement. The computer vision method tested on the same sample as the fruit battery method shows lower accuracy score compared to the fruit battery method. On the other hand, by combining both features, the accuracy score increased to 94.2%.

The combination method proved to be helpful, since the fruit battery method detects the change in the fruit’s chemistry, and the computer vision using color feature detects the changes in color due to changing chlorophyll and anthocyanin content on the fruit’s surface [8]. From this study, it is shown that the combined feature can classify the oil palm fruit maturity stages with higher accuracy compared to one-dimensional features.

**Figure 8:**
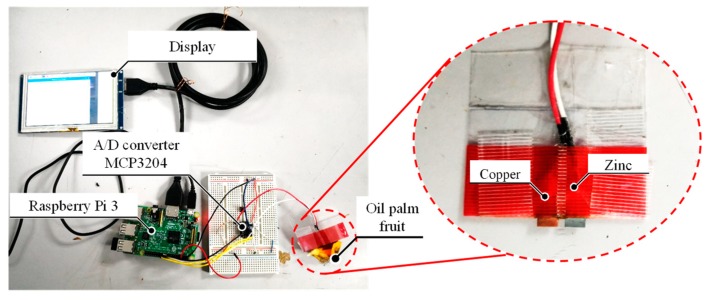
The prototype device that estimates oil palm fruit’s moisture content using Raspberry Pi 3 Model B.

Based on the results obtained from the experiments, a fruit battery prototype to test the oil palm fruit maturity was fabricated using open source hardware Raspberry Pi 3 Model B as shown in Figure 8. The 12-bit A/D converter was connected to Raspberry Pi 3 Model B with 0.8 mV A/D converter voltage resolution and 3.3 V drive voltage.

From Figure 4, the computer vision method used in this study involved an AR color chart, and the sample was taken together in one photo. However, this method is prone to inconsistency and error due to lighting, type of camera, camera operating setting as well as the photo compression and so forth, where the color may not be consistent and accurately captured by device in comparison to real life. Hence, for further future improvement for computer vision, it is crucial to apply color calibration in order to determine the accuracy of the image data collected. The color calibration RPS-3D colorimetric shows the ability to reduce the environmental effect that is essential in analyzing data that involves camera vision [18]. Besides that, in order to improve the data accuracy, the number of samples needs to be bigger for better sample population representation.

## 5. Conclusions

This research studied the fruit battery load resistance determination that produces low resolution for high-sensitivity results. According to the results, the best load resistance obtained is 1 kΩ with high changing rate between unripe and ripe fruit at 74% and moisture content resolution at 0.517%/mV. The accuracy scores for fruit battery and computer vision are 90.4% and 86.5%, respectively. By combining the fruit battery and computer vision methods, the calculated accuracy score increased to 94.2%. Since this method is simple and cheap, it can be an additional or alternative source of oil palm fruit maturity checking in the oil palm mill during inspection as well as during harvesting. Regardless, this research opens up a new study for oil palm fruit ripeness classification methods.

## Figures and Tables

**Table 1:** Oil palm fruit ripeness based on moisture content.

Ripeness Category	Moisture Content
Ripe	<30%
Under-ripe	30–53%
Unripe	>53%

**Table 2:** Experimental setup.

Item	Type/Value
Electrode material	Zinc, copper
Electrode dimension	16 mm × 6 mm × 0.5 mm
Distance between electrodes	2 mm
Depth of electrodes	3 mm
Load resistance, *R*_L_ (Ω)	10, 100, 1 k, 10 k, 100 k, 1 M

**Table 3:** Classification condition setting for support vector machine (SVM).

Item	Type/Value
Feature	Fruit battery: *V*_L_
	Computer vision: R_ave_/G_ave_
	Combination: *V*_L_-R_ave_/G_ave_
Score	Accuracy
Cost parameter	1, 10, 100
Gamma	1, 0.1, 0.01
Kernel	Linear, rbf
Number of partitions of k-fold cross validation	8

**Table 4:** Accuracy score and standard deviation of each feature for fruit battery method.

Cost Parameter	Gamma	Kernel	Accuracy Score	Standard Deviation
1	1	linear	0.7308	0.1028
1	1	rbf	0.9038	0.0712
1	0.1	linear	0.7308	0.1028
1	0.1	rbf	0.6923	0.079
1	0.01	linear	0.7308	0.1028
1	0.01	rbf	0.6923	0.079
10	1	linear	0.8269	0.1471
10	1	rbf	0.8846	0.0648
10	0.1	linear	0.8269	0.1471
10	0.1	rbf	0.9038	0.0712
10	0.01	linear	0.8269	0.1471
10	0.01	rbf	0.6923	0.079
100	1	linear	0.8462	0.109
100	1	rbf	0.9038	0.0766
100	0.1	linear	0.8462	0.109
100	0.1	rbf	0.8846	0.0648
100	0.01	linear	0.8462	0.109
100	0.01	rbf	0.8077	0.1334

**Table 5:** Accuracy score and standard deviation of each feature for computer vision method.

Cost Parameter	Gamma	Kernel	Mean	Standard Deviation
1	1	linear	0.6538	0.1042
1	1	rbf	0.8654	0.0925
1	0.1	linear	0.6538	0.1042
1	0.1	rbf	0.6538	0.1042
1	0.01	linear	0.6538	0.1042
1	0.01	rbf	0.6346	0.0959
10	1	linear	0.6538	0.1042
10	1	rbf	0.8269	0.1263
10	0.1	linear	0.6538	0.1042
10	0.1	rbf	0.6731	0.1413
10	0.01	linear	0.6538	0.1042
10	0.01	rbf	0.6538	0.1042
100	1	linear	0.6538	0.1042
100	1	rbf	0.8269	0.1263
100	0.1	linear	0.6538	0.1042
100	0.1	rbf	0.8269	0.1429
100	0.01	linear	0.6538	0.1042
100	0.01	rbf	0.6538	0.1042

**Table 6:** Accuracy score and standard deviation of each feature for the combination of fruit battery and computer vision method.

Cost Parameter	Gamma	Kernel	Mean	Standard Deviation
1	1	linear	0.75	0.0959
1	1	rbf	0.9038	0.1204
1	0.1	linear	0.75	0.0959
1	0.1	rbf	0.75	0.0959
1	0.01	linear	0.75	0.0959
1	0.01	rbf	0.6923	0.0419
10	1	linear	0.8654	0.1266
10	1	rbf	0.8654	0.1289
10	0.1	linear	0.8654	0.1266
10	0.1	rbf	0.9423	0.0804
10	0.01	linear	0.8654	0.1266
10	0.01	rbf	0.6923	0.0419
100	1	linear	0.8846	0.0965
100	1	rbf	0.8462	0.1575
100	0.1	linear	0.8846	0.0965
100	0.1	rbf	0.9231	0.1101
100	0.01	linear	0.8846	0.0965
100	0.01	rbf	0.8654	0.1248

**Table 7:** Maximum accuracy score and standard deviation of each feature.

Feature	Accuracy (%)	Standard Deviation
Fruit battery method using load resistance voltage, *V*_L_	90.4	0.0712
Computer vision using color feature, R_ave_/G_ave_	86.5	0.0925
Combined feature (fruit battery and computer vision), *V*_L_-R_ave_/G_ave_	94.2	0.0804
